# Supplementary material for: Quantifying Plasmodium falciparum infections clustering within households to inform household-based intervention strategies for malaria control programs: An observational study and meta-analysis from 41 malaria-endemic countries
Source: PLoS Med. 2020 Oct 29;17(10):e1003370. doi: 10.1371/journal.pmed.1003370 (PMC7595326; doi:10.1371/journal.pmed.1003370)
Supplement: S1 Table — (DOCX) [file pmed.1003370.s001.docx]

**Supporting Table 1: Definition and Extraction of Treatment Seeking Variables**

| **Variable Label** | **Description** | **Response** | **Survey Usage** |
| --- | --- | --- | --- |
| h37a | Fansidar taken for fever/cough | 0 no, 1 yes, 9 missing | All Where Used |
| h37aa | Artesunate rectal taken for fever | 0 no, 1 yes, 9 missing | All Where Used |
| h37ab | Artesunate injection/IV taken for fever | 0 no, 1 yes, 9 missing | All Where Used |
| h37b | Chloroquine taken for fever/cough | 0 no, 1 yes, 9 missing | All Where Used |
| h37c | Amodiaquine taken for fever/cough | 0 no, 1 yes, 9 missing | All Where Used |
| h37d | Quinine taken for fever/cough | 0 no, 1 yes, 9 missing | All Where Used |
| h37da | Quinine injection/IV taken for fever | 0 no, 1 yes, 9 missing | All Where Used |
| h37e | Combination with artemisinin taken for fever/cough | 0 no, 1 yes, 9 missing | All Where Used |
| h37f | CS antimalarial taken for fever/cough | 0 no, 1 yes, 9 missing | All Where Used |
| h37g | (CS) PRIMO taken for fever/cough | 0 no, 1 yes, 9 missing | All Where Used |
| h37h | Other antimalarial taken for fever/cough | 0 no, 1 yes, 9 missing | All Where Used |
| h37n | Artesunate mefloquine taken for fever | 0 no, 1 yes, 9 missing | 53 only |
| ml13a | Fansidar taken for fever | 0 no, 1 yes, 9 missing | All Where Used |
| ml13aa | Artesunate taken for fever | 0 no, 1 yes, 9 missing | All Where Used |
| ml13ab | Artesunate injection/IV taken for fever | 0 no, 1 yes, 9 missing | All Where Used |
| ml13b | Chloroquine taken for fever | 0 no, 1 yes, 9 missing | All Where Used |
| ml13c | Amodiaquine taken for fever/cough | 0 no, 1 yes, 9 missing | All Where Used |
| ml13d | Quinine taken for fever | 0 no, 1 yes, 9 missing | All Where Used |
| ml13da | Quinine injection/IV taken for fever | 0 no, 1 yes, 9 missing | All Where Used |
| ml13e | Combination with artemisinin taken for fever/cough | 0 no, 1 yes, 9 missing | All Where Used |
| ml13f | Coartem taken for fever/cough | 0 no, 1 yes, 9 missing | All Where Used |
| ml13g | AL/Artemether Lumefantrine taken for fever | 0 no, 1 yes, 9 missing | All Where Used |
| ml13h | Other antimalarial taken for fever | 0 no, 1 yes, 9 missing | All Where Used |
| ml13n | Artesunate ( injection ) taken for fever/cough | 0 no, 1 yes, 9 missing | 17, 53 Only |
| s623a | Medicine taken during fever: Antimalarial, combination with artemisinin (tca) | 0 no, 1 yes, 8 dk, 9 missing | All Where Used |
| s623b | Medicine taken during fever: Antimalarial, Sp/fansidar | 0 no, 1 yes, 8 dk, 9 missing | All Where Used |
| s623c | Medicine taken during fever: Antimalarial, Chloroquine | 0 no, 1 yes, 8 dk, 9 missing | All Where Used |
| s623d | Medicine taken during fever: Antimalarial, Amodiaquine | 0 no, 1 yes, 8 dk, 9 missing | All Where Used |
| s623e | Medicine taken during fever: Antimalarial, Quinine pills | 0 no, 1 yes, 8 dk, 9 missing | All Where Used |
| s623f | Medicine taken during fever: Antimalarial, Quinino injection | 0 no, 1 yes, 8 dk, 9 missing | All Where Used |
| s623g | Medicine taken during fever: Antimalarial, Coartem | 0 no, 1 yes, 8 dk, 9 missing | All Where Used |
| s623h | Medicine taken during fever: Antimalarial, Other | 0 no, 1 yes, 8 dk, 9 missing | All Where Used |
| s538c | Dihidroartemis- Piperaquine taken for fever | 0 no, 1 yes, 9 missing | All Where Used |
| s538e | Artesunate-Amodiaquine taken for fever | 0 no, 1 yes, 9 missing | All Where Used |
| s538g | Malaxin taken for fever/cough | 0 no, 1 yes, 9 missing | All Where Used |
| s538h | Malaritab taken for fever/cough | 0 no, 1 yes, 8 dk, 9 missing | All Where Used |
| s538i | Arinate taken for fever/cough | 0 no, 1 yes, 8 dk, 9 missing | All Where Used |
| s538j | Artesunate taken for fever/cough | 0 no, 1 yes, 8 dk, 9 missing | All Where Used |
| s538k | Mefloquine taken for fever/cough | 0 no, 1 yes, 8 dk, 9 missing | All Where Used |
| s125a | Antimalaria medicines prescribed or taken: SP/Sulphadoxine Pyrimethamine | 0 no, 1 yes, 9 missing | All Where Used |
| s125b | Antimalaria medicines prescribed or taken: chloroquine | 0 no, 1 yes, 9 missing | All Where Used |
| s125c | Antimalaria medicines prescribed or taken: DP/Dihydroartemisinin-Piperaquine | 0 no, 1 yes, 9 missing | All Where Used |
| s125d | Antimalaria medicines prescribed or taken: quinine | 0 no, 1 yes, 9 missing | All Where Used |
| s125e | Antimalaria medicines prescribed or taken: AA/Artesunate Amodiaquine | 0 no, 1 yes, 9 missing | All Where Used |
| s125f | Antimalaria medicines prescribed or taken: artemisinin | 0 no, 1 yes, 9 missing | All Where Used |
| s125g | Antimalaria medicines prescribed or taken: AL/Artemether-Lumefantrine | 0 no, 1 yes, 9 missing | All Where Used |
| s412a | Combination with artemisinin taken for fever/cough | 0 no, 1 yes, 9 missing | All Where Used |
| s412b | Fansidar taken for fever/cough | 0 no, 1 yes, 9 missing | All Where Used |
| s412c | Chloroquine taken for fever/cough | 0 no, 1 yes, 9 missing | All Where Used |
| s412d | Amodiaquine taken for fever/cough | 0 no, 1 yes, 9 missing | All Where Used |
| s412e | Quinine pill taken for fever/cough | 0 no, 1 yes, 9 missing | All Where Used |
| s412f | Quinine injection taken for fever/cough | 0 no, 1 yes, 9 missing | All Where Used |
| s412g | Artesunate : by rectal taken for fever/cough | 0 no, 1 yes, 9 missing | All Where Used |
| s412h | Artesunate : by injection taken for fever/cough | 0 no, 1 yes, 9 missing | 15, 28 Only |
| s412i | Fansidar and Amodiaquine (combined) taken for fever/cough | 0 no, 1 yes, 9 missing | All Where Used |
| s412j | Other antimalarial taken for fever/cough | 0 no, 1 yes, 9 missing | 28 Only |
| s326f | Larimal taken for fever/cough | 0 no, 1 yes, 8 dk, 9 missing | All Where Used |
| s326h | Arsumoon taken for fever/cough | 0 no, 1 yes, 8 dk, 9 missing | All Where Used |
| s326i | Falcimon taken for fever/cough | 0 no, 1 yes, 8 dk, 9 missing | All Where Used |
| s326j | Asaq Wintrop taken for fever/cough | 0 no, 1 yes, 8 dk, 9 missing | All Where Used |
| s326k | Artefan taken for fever/cough | 0 no, 1 yes, 8 dk, 9 missing | All Where Used |
| s411a | Actipal taken for fever | 0 no, 1 yes, 9 missing | All Where Used |
| s411b | Larimal taken for fever | 0 no, 1 yes, 9 missing | All Where Used |
| s411c | Artemodi taken for fever | 0 no, 1 yes, 9 missing | All Where Used |
| s411d | Arsumoon taken for fever | 0 no, 1 yes, 9 missing | All Where Used |
| s411e | Falcimon taken for fever | 0 no, 1 yes, 9 missing | All Where Used |
| s411f | Other ASAQ taken for fever | 0 no, 1 yes, 9 missing | All Where Used |
| s411g | Quinine (injection/IV) taken for fever/cough | 0 no, 1 yes, 9 missing | All Where Used |
| s411h | Artefan taken for fever | 0 no, 1 yes, 9 missing | All Where Used |
| s411i | Lumartem taken for fever | 0 no, 1 yes, 9 missing | All Where Used |
| s411j | Other AL taken for fever | 0 no, 1 yes, 9 missing | All Where Used |
| s411asaq | Any ASAQ taken for fever | 0 no, 1 yes, 9 missing | All Where Used |
| s411al | Any AL taken for fever | 0 no, 1 yes, 9 missing | All Where Used |
| s124 | Any antimalaria medicine prescribed to treat the malaria or took medicines without prescription | 0 no, 1 yes, prescribed, 2, yes took without prescription | All Where Used |
| s311a | Fansidar taken for fever | 0 no, 1 yes | All Where Used |
| s311b | Chloroquine taken for fever | 0 no, 1 yes | All Where Used |
| s311c | Amodiaquine taken for fever | 0 no, 1 yes | All Where Used |
| s311d | Quinine taken for fever | 0 no, 1 yes | All Where Used |
| s311e | Combination with artemisinin taken for fever | 0 no, 1 yes | All Where Used |
| s311f | Coartem taken for fever | 0 no, 1 yes | All Where Used |
| s311g | Other antimalarial taken for fever | 0 no, 1 yes | All Where Used |
| s623a | Medicine taken during fever: Antimalarial, combination with artemisinin (tca) | 0 no, 1 yes, 8 dk | All Where Used |
| s623b | Medicine taken during fever: Antimalarial, Sp/fansidar | 0 no, 1 yes, 8 dk | All Where Used |
| s623c | Medicine taken during fever: Antimalarial, Chloroquine | 0 no, 1 yes, 8 dk | All Where Used |
| s623d | Medicine taken during fever: Antimalarial, Amodiaquine | 0 no, 1 yes, 8 dk | All Where Used |
| s623e | Medicine taken during fever: Antimalarial, Quinine pills | 0 no, 1 yes, 8 dk | All Where Used |
| s623f | Medicine taken during fever: Antimalarial, Quinino injection | 0 no, 1 yes, 8 dk | All Where Used |
| s623g | Medicine taken during fever: Antimalarial, Coartem | 0 no, 1 yes, 8 dk | All Where Used |
| s623h | Medicine taken during fever: Antimalarial, Other | 0 no, 1 yes, 8 dk | All Where Used |
| **Note:** Sometimes the variable descriptions are different across surveys despite the same Variable Label. We went through all of the Variable Labels individually to assess. In instances where all different versions of the label still pertained to treatment seeking behaviour, the label was retained and used in all surveys (denote "All Where Used" in Survey Usage). Where only a subset of the label occurrences pertained to malaria treatment seeking, the variable was used only for a subset of surveys, with that information detailed in the "Survey Usage" column. | | | |
