# Supplementary material for: Quantifying Plasmodium falciparum infections clustering within households to inform household-based intervention strategies for malaria control programs: An observational study and meta-analysis from 41 malaria-endemic countries
Source: PLoS Med. 2020 Oct 29;17(10):e1003370. doi: 10.1371/journal.pmed.1003370 (PMC7595326; doi:10.1371/journal.pmed.1003370)
Supplement: S2 Table — (DOCX) [file pmed.1003370.s002.docx]

| **Supporting Table 2: Results of the Logistic Regression Modelling For Each of the Three Models Assessed using the DHS Datasets.** Separate logistic regression models were fitted relating an individual’s malaria positivity to survey prevalence, household size and one of three factors related to the status of other individuals’ resident in the household. An interaction term between this other factor and survey prevalence was also included in the model to allow this relationship to vary with survey prevalence. These models were fitted to the data within a Bayesian framework using the probabilistic programming language STAN, and implemented in R using the package RStan. Results presented below are based on 10,000 HMC samples. | | | | |
| --- | --- | --- | --- | --- |
| **Model** | **Survey Prevalence** | **Household Size** | **Index Household** | **Interaction** |
| **RACD - Community** | 1.06 (1.06 - 1.06) | 1.00 (0.99 - 1.01) | 11.72 (10.42 - 13.19) | 0.96 (0.96 - 0.97) |
| **MSAT** | 1.06 (1.06 - 1.06) | 0.98 (0.97 - 0.99) | 14.17 (13.09 - 15.32) | 0.97 (0.97 - 0.97) |
| **MTAT** | 1.05 (1.05 - 1.05) | 0.88 (0.87 - 0.88) | 20.41 (19.36 - 21.53) | 0.98 (0.97 - 0.98) |
