# Supplementary material for: Quantifying Plasmodium falciparum infections clustering within households to inform household-based intervention strategies for malaria control programs: An observational study and meta-analysis from 41 malaria-endemic countries
Source: PLoS Med. 2020 Oct 29;17(10):e1003370. doi: 10.1371/journal.pmed.1003370 (PMC7595326; doi:10.1371/journal.pmed.1003370)
Supplement: S1 Text — (DOCX) [file pmed.1003370.s003.docx]

**Supporting Text 1: Detailed Methods for DHS Data Extraction**

**Data Extraction, Collation and Pre-Analysis Processing**

Below is a detailed description of the workflow responsible for generating the dataset used in the analyses presented in this paper. Briefly, it describes the steps involved in the identification of relevant DHS surveys, extraction of data from these surveys, and the downstream pre-processing carried out on the extracted data before statistical analyses were conducted.

**1. Extract People’s Recode and Children’s Recode For Each Survey**

The Demographic and Health Surveys (DHS) Program is an organisation responsible for collecting and disseminating accurate, nationally representation data on health and population in developing countries. This data is collected through conducting surveys involving in-depth interviews of households and individuals, following a set methodology and using a routine questionnaire consisting of core questions (collecting a suite of information on various economic, health-related and demographic variables) that are supplemented with a variety of different questions depending on the context and particular survey. The results of these surveys are then processed into different datasets (called “recodes”) characterised by different units of analysis (broadly, “who” or “what” is being studied). Depending on the recode, these units of analysis are typically either households, women, children or men. Each recode typically contains a core set of uniquely identifying information (Age, Sex, Region etc) that is present across all recodes, as well as information that is unique to that recode (and present in that recode only).

Using the recently developed rDHS package, we identified all surveys that had assessed malaria infection status by either rapid diagnostic test (RDT, SurveyCharacteristicID 89 in the rDHS package) or light microscopy (SurveyCharacteristicID 90 in the rDHS package). In total, 56 surveys were identified in this way, as well as a single additional survey not reporting SurveyCharacteristicID 89 or 90 but where malaria infection status had been ascertained. Together, these 57 surveys spanned the years 2006 – 2018, and included over 2.5 million individuals (although the number included in our analyses is far lower as only a subset of these individuals had their malaria status tested, see below for further details) from 23 countries across sub-Saharan Africa.

**2. Exclude Individuals Not Tested for Malaria and Those Over 7 Years Old**

Of the surveys identified, 51 tested for malaria infection only in children under 7, whilst the remaining tested all individuals regardless of age. The outcome of interest in the analyses conducted here was the probability of an individual being infected or not, and so individuals not tested for malaria were excluded. Many of the variables of interest (recent fever occurrence and the treatment seeking variables used to define treatment seeking behaviour) were only available for children under 7 (and missing otherwise) and so individuals >7 years were also excluded from the analyses presented in the main text. Our primary dataset for consideration therefore consists of children under 7 whose malaria status had been determined by either RDT or microscopy and for whom information was available on recent fever occurrence and treatment seeking behaviour. This information (on malaria status, recent fever occurrence, treatment seeking status etc) is not contained in a single part each of Demographic Health Survey. Instead, this information was split across two datasets associated with each survey: the “People’s Recode” (containing information on malaria infection status) and the “Children’s Recode” (containing information on recent fever occurrence and treatment seeking behaviour).

**3. Linkage of Individuals Across Survey Recodes**

The variables of interest required for our analyses were split across different survey recodes, necessitating linkage of individuals across both datasets. In order to do this, we extracted uniquely identifying information that was present and detailed across both survey recodes. Specifically, these variables were Age, Sex, Cluster Number, Household Number and Line Number. Using these variables, we were able to link the vast majority of respondents (consistently >90%) across both datasets (see Supplementary Information *Survey Information*), allowing near complete collation of malaria infection status, recent fever occurrence and treatment seeking behaviour across surveyed individuals. Inability to link all individuals likely arises either from errors associated with individual enumeration of data entry or issues surrounding who is included in the “Children’s Recode”. Specifically, if a child in the household during the survey is without a mother (who would typically be interviewed to provide data for the “Children’s Recode”), that child will not be present in the “Children’s Recode” but will be present in the “Person’s Recode”. Irrespective, these instances represented a small fraction of the overall individuals surveyed and are therefore unlikely to affect the conclusions arising from subsequent analysis of the data.

**4. Variable Definition and Generation**

Infection status was detected by either RDT (n = 11) or Light Microscopy and RDT (n = 45). There was one survey for which it was unclear which technique had been used. Malaria infection status was typically coded using the variables *hml32* (for Light Microscopy testing results) and *hml35* (for RDT testing results) from the People’s Recode. There were a small number of instances where different variable labels were used (n = 4). In these instances, the relevant, survey specific variable denoting malaria status were used instead (see Supplementary Information *Survey Information* for further details). Individuals were defined as positive if the individual tested positive for malaria by either method.

Recent fever occurrence was defined based on the survey variable *h22* from the Children’s Recode, which describes whether the child has experienced fever during the past 2 weeks. To characterise treatment seeking behaviour, we examined the full array of malaria treatment related variables detailed in each survey’s Children’s Recode, utilising them as a proxy for treatment seeking behaviour (i.e. we assume that an individual receiving a particular anti-malarial treatment recently is indicative of that individual having recently sought treatment). Although treatment related variables were largely consistent across surveys, there were a number of survey-specific treatment related variables, asked as an additional, non-standard question during the survey. For the analyses presented here, we utilised all of the treatment-seeking variables available for a given survey, with an individual defined as having sought treatment if any one of these extracted variables was positive. For a full list of the variables used to define treatment-seeking behaviour for each survey, see Supplementary Information *Treatment Seeking Variables*. In addition to these variables, we also extracted uniquely identifying information for each individual, including their region of residence, their household number (as enumerated by the survey) and unique line number, allowing linkage of children between the People’s Recode (containing information of malaria status) and the Children’s Recode (containing information on recent fever occurrence and treatment seeking behaviour) associated with each survey.

Using these collated variables, we defined a set of three binary indicators for each individual that describe whether that individual belongs to an Index household, as defined each of the programmatic strategies being considered. Briefly, an individual was defined as residing in an index household if the household contained at least one other infection detectable through one of the programmatic strategies considered i.e. through i. clinical care seeking (RACD model), ii. through household surveys using routine diagnostics in symptomatic individuals (MSAT) or regardless of symptoms (iii. MTAT). For a given individual, these binary indicators describing whether that individual resides in an Index Household are defined in the following way:

- ***MTAT Index Household Status (MTAT)*:** 1 if any other child in the household is malaria positive and 0 otherwise (i.e. all other children in the household who are tested are malaria negative).
- ***MSAT Index Household Status (MSAT)*:** 1 if any other child in the household has had a malaria-related fever during the past two weeks (defined as that other child in the household being malaria positive and having had a fever during the past 2 weeks) and 0 otherwise (i.e. all other children in the household who were tested and provide responses are malaria negative and have not reported a fever during the past 2 weeks respectively).
- ***RACD Index Household Status (RACD)*:** 1 if any other child in the household is positive for malaria and has sought anti-malarial treatment (based on the collated treatment seeking variables), and 0 otherwise (i.e. all other children in the household who are tested and provide responses are malaria negative and have not sought anti-malaria treatment respectively).

This resulted in 3 datasets containing identical information except for the binary indicator (which differs between datasets). This separation was done as many more individuals had been tested for malaria than reported recent fever occurrence or treatment seeking status and so defining the 3 separately enabled us to maximise the number of individuals included in each dataset. This means that each dataset contains all individuals for whom malaria status was determined and it was possible to define Index household status for. Definition of Index Household status in this way is contingent upon a household containing multiple individuals, and so we implicitly remove all children who are the sole respondent detailed in the surveys. For full details on the number of children for whom these variables could be defined, and by extension, the overall size of the datasets, see Supplementary Information *Survey Information and Details*.

**Note:** In a small number of surveys (n = 6), malaria status had been assessed across all ages. Because of this, it was possible to define Index Household status for individuals aged greater than 7 years, due to their sharing a household with a child under 7 containing complete, positive information on malaria infection status, recent fever occurrence and treatment seeking behaviour (see below for further information on how these binary indicators were defined). We therefore replicated the analyses presented in the main text retaining these individuals (spanning a wider range of ages), although their inclusion did not qualitatively alter the conclusions drawn (Supplementary Figure A).

**Note:** The definition used for Index Household in the DHS analyses is based on other members of the household (i.e. not including the individual being considered) and whether they represent infections detectable according to one of the three programmatic criteria. This is subtly different from the definition used to define Index Households in the analysis of the published literature, where Index Household status is defined based on all members of the household (including the individual being considered).

The difference in these approaches to Index Household definition were due to limitations surrounding the DHS data. Specifically, the DHS surveys only tested for malaria using routine diagnostics (Microscopy or RDTs) – the result of this is that they only report patent infections. This posed a problem for evaluating the clustering of infections according to the MTAT strategy – the lack of molecular diagnosis (of subpatent infections) means all individuals positive for malaria in the DHS surveys (representing only patent infections) would be in “Index” households. Because of this constraint, when analysing the DHS data, we adopted the alternative definition of Index Households given above.

However, during analysis of the DHS data, it was possible to evaluate the RACD and MSAT approaches using the same Index Household definition used for the published literature. The results of this are presented in Supplementary Figure B and qualitatively support the results presented in Figure 1 of the main text. This suggests that our observation of increased household clustering of programmatically detectable malaria infections at low transmission is robust to the different Index Household definitions utilised.


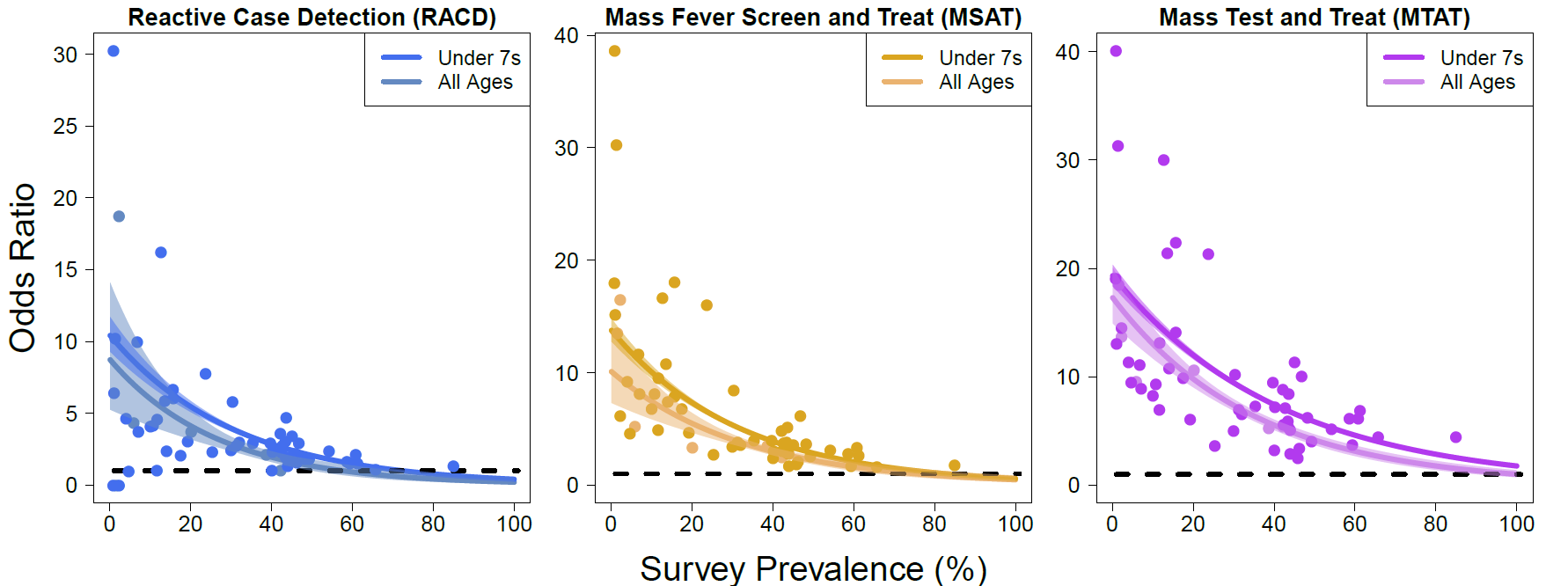


**Supplementary Figure A:** **Malaria infection and the extent of household clustering according to detectability by different programmatic strategies in all age surveys compared to children under 7 only.** Data from the 51 DHS surveys in which only children under 7 had been tested for malaria infection (dark line with corresponding 95% credible interval in shaded area), and the 6 surveys in which all ages had been tested (lighter line with corresponding 95% credible interval in shaded area) were extracted and analysis repeated to compare the trends with the few all age surveys with the results from the larger dataset with children under 7 years of age. The dashed line represents an Odds Ratio (OR) of 1 with the dots representing the empirically estimated OR from each DHS study.


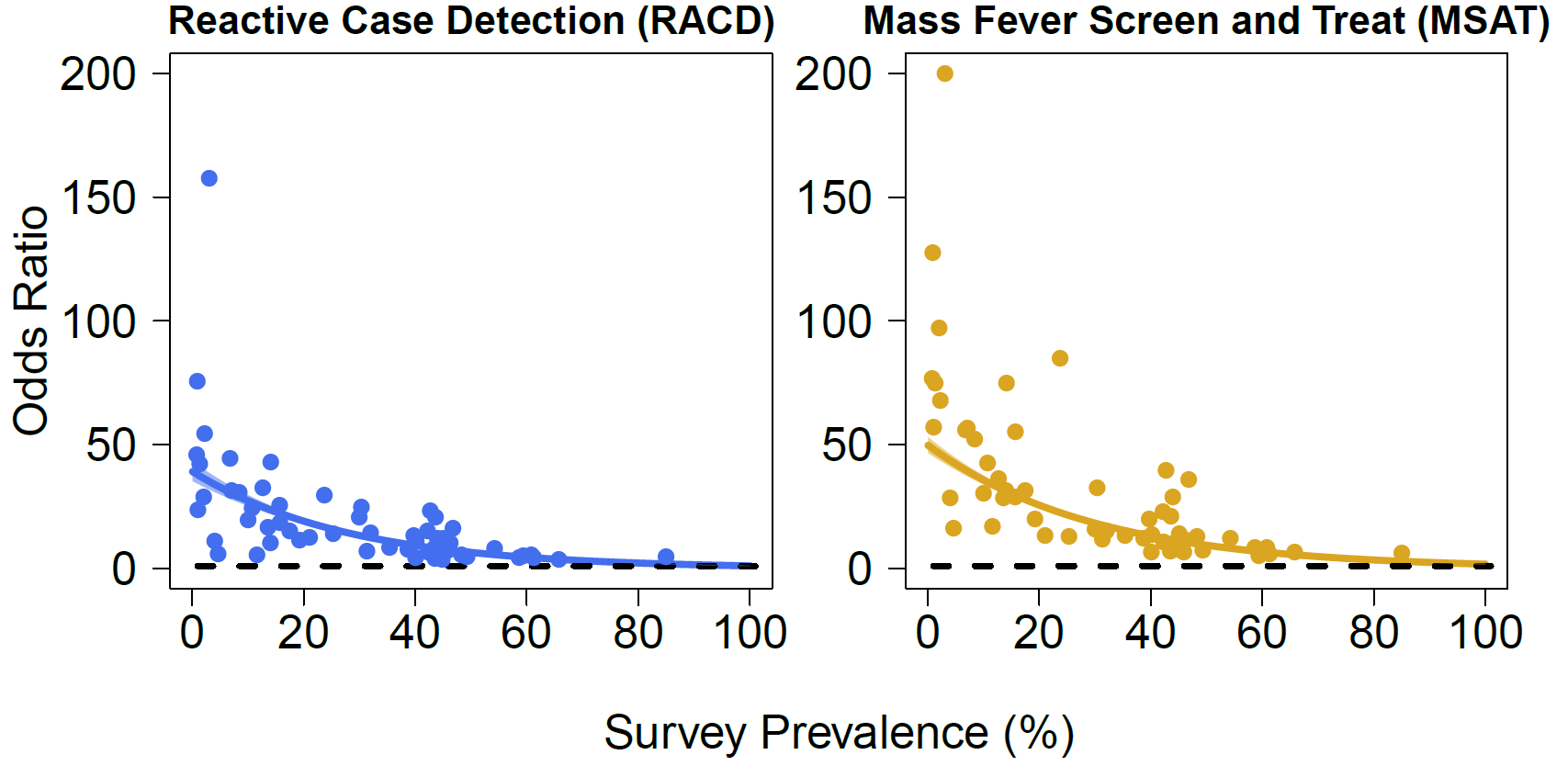


**Supplementary Figure B: Malaria infection and the extent of household clustering according to detectability by different programmatic strategies** **using an analogous definition to that used in the meta-analysis.** Due to limitations in the DHS data (specifically that only routine diagnostics rather than molecular methods had been used to diagnose infection), only patent infections were detected and recorded. This constraint necessitated adopting a slightly different definition of Index Households in the DHS analysis compared to the analyses of the published literature. Whilst not possible to evaluate the MTAT strategy using the DHS data and the Index Household definition utilised for the published literature, it was possible to do so for RACD and MSAT. These results are presented above, where the line represents the modelled OR of an individual being malaria positive given they reside in an Index Household (using the same definition as in the analyses of the published literature) compared to those who do not reside in an Index Household, with corresponding 95% credible interval in shaded area. The dashed line represents an Odds Ratio (OR) of 1 with the dots representing the empirically estimated OR from each DHS study.
