# Supplementary material for: Quantifying Plasmodium falciparum infections clustering within households to inform household-based intervention strategies for malaria control programs: An observational study and meta-analysis from 41 malaria-endemic countries
Source: PLoS Med. 2020 Oct 29;17(10):e1003370. doi: 10.1371/journal.pmed.1003370 (PMC7595326; doi:10.1371/journal.pmed.1003370)
Supplement: S2 Text — (DOCX) [file pmed.1003370.s004.docx]

**Supporting Text 2: Statistical Analysis and Regression Modelling of DHS Data**

To this collated data, we fitted a logistic regression model relating malaria infection status to the collated variables This model was fitted within a Bayesian framework, with parameter inference carried out using a Markov Chain Monte Carlo (MCMC) based sampling scheme implemented in the probabilistic programming language STAN. Uninformative priors (Normal distributions with mean 0 and standard deviation 15) were set for all inferred parameters.

Specifically, we assume malaria infection status (a binary indicator taking either 0 or 1, indicating malaria absence and presence respectively) for individual $i$, belonging to household $j$ surveyed as part of survey $k$ to be drawn from a Bernoulli distribution:

$${Malaria Infectious Status}_{i, j, k} \sim Bernoulli({Prob Malaria Positive}_{i,j, k})$$

where ${Prob Malaria Positive}_{i,j, k}$ represents the probability of that individual being malaria positive. We model this probability using logistic regression i.e. as being a linear function of a number of different covariates on the logit scale. It is specified in the following way:

$logit\left( {Prob Malaria Positive}_{i,j,k} \right)= \alpha_{k}+ \beta_{1}{Index Household}_{i, j, k}+\beta_{2}{Prevalence}_{k}+ \beta_{3}{Household Size}_{j,k}+ + \beta_{4}({Prevalence}_{k}*{Index Household}_{i,j,k}$)

where:

- ${Index Household}_{i, j, k}$ is one of the three binary indicators (either 0 or 1) for individual $i$, belonging to household $j$ surveyed as part of survey $k$ describing one of three following scenarios:
  - Whether anyone else in the individual’s household is malaria positive (i.e. the individual resides in an Index Household as defined by the MTAT approach).
  - Whether anyone else in the individual’s household is malaria positive and has had fever in the past 2 weeks (i.e. the individual resides in an Index Household as defined by the MSAT approach).
  - Whether anyone else in the individual’s household is malaria positive and has sought treatment for malaria (i.e. the individual resides in an Index Household as defined by the RACD approach).
- ${Prevalence}_{k}$ is the overall malaria prevalence recorded for survey $k$.
- ${Household Size}_{j,k}$ is the size of household $j$ in survey $k$ where individual $i$ is resident.
- $\beta_{1-4}$ are regression coefficients determining the magnitude of the influence of each variable on the probability of being malaria positive. Individually, they describe the odds ratio of being malaria positive vs malaria negative for a unit increase (for continuous variables) or an indicator is 1 (rather than 0, for binary variables), all other variables kept equal.
- $\alpha_{k}$ is an intercept within the regression framework used.
- ${Prevalence}_{k}*{Index Household}_{i,j,k}$ is an interaction term describing how the effect of ${Index Household}_{i,j,k}$ varies with the overall survey prevalence (a proxy for overall transmission intensity).

Our primary interest is to assess how the influence of the three binary indicators detailing Index Household residency ($Index Household$) vary with transmission intensity (described by the survey prevalence $Prevalence$) and so we include an interaction term involving these two variables. This allows the influence of $Index Household$ to vary with survey prevalence, allowing us to formally test whether the odds ratio associated with $Index Household$differs systematically according to transmission intensity – this formally tests the hypothesis that the association between being malaria positive and sharing a household with someone who satisfies one of the three programmatic criteria (Malaria Positivity for MTAT, Recent Malaria Fever for MSAT or Malaria and Treatment Seeking Behaviour for RACD) varies across the transmission spectrum (i.e. Prevalence). It therefore explores the extent to which these individuals cluster and how this varies depending on the level of malaria endemicity.

We fitted three separate instances of this model, using each of the three binary indicators individually. This framework was written in the probabilistic programming language STAN, which employs a gradient-based MCMC algorithm (the No-U-Turn sampler, a variant of Hamiltonian Monte Carlo) for Bayesian Inference, and implemented in R using the rSTan package. A total of 5 chains, each 4,000 iterations in length, were run for purposes of model fitting and parameter inference. Half of each chain’s iterations were discarded as burn-in/the adaptive phase of the sampling, leaving a total of 10,000 iterations available for inference. Measures of MCMC convergence such as the Gelman-Rubin statistic were monitored and were all consistently <1.02, indicating stability of the chains and supporting the probability of convergence to the underlying true posterior distribution. Details of the exact STAN code used to run the regression analyses for each survey is given below.

**STAN Code for the Model Used**

**data {**

int<lower=0> N;

vector[N] Overall_Prevalence;

vector[N] Other_HH_Member_Status;

vector[N] Num_Respondents;

int Infection_Status[N];

**}**

**transformed data {**

vector[N] interaction;

interaction = Overall_Prevalence .* Other_HH_Member_Status;

**}**

**parameters {**

real intercept;

real overall_prev_coef;

real other_hh_member_coef;

real interaction_coef;

real num_resp_coef;

**}**

**model {**

intercept ~ normal(0, 15);

overall_prev_coef ~ normal(0, 15);

other_hh_member_coef ~ normal(0, 15);

interaction_coef ~ normal(0, 15);

num_resp_coef ~ normal(0, 15);

Infection_Status ~ bernoulli_logit(intercept + overall_prev_coef * Overall_Prevalence + other_hh_member_coef * Other_HH_Member_Status + interaction_coef * interaction + num_resp_coef * Num_Respondents);

**}**
