# Supplementary material for: Quantifying Plasmodium falciparum infections clustering within households to inform household-based intervention strategies for malaria control programs: An observational study and meta-analysis from 41 malaria-endemic countries
Source: PLoS Med. 2020 Oct 29;17(10):e1003370. doi: 10.1371/journal.pmed.1003370 (PMC7595326; doi:10.1371/journal.pmed.1003370)
Supplement: S4 Text — (DOCX) [file pmed.1003370.s006.docx]

**Supporting Text 4: Reference List for Published Studies Included in the Meta-Analysis**

^1-35^

1. Tripura R, Peto TJ, Chalk J, et al. Persistent Plasmodium falciparum and Plasmodium vivax infections in a western Cambodian population: implications for prevention, treatment and elimination strategies. *Malaria journal* 2016; **15**: 181.

2. Tripura R, Peto TJ, Nguon C, et al. A Controlled Trial of Mass Drug Administration to Interrupt Transmission of Multi Drug Resistant Falciparum Malaria in Cambodian Villages. *Clinical infectious diseases : an official publication of the Infectious Diseases Society of America* 2018.

3. Parker DM, Tripura R, Peto TJ, et al. A multi-level spatial analysis of clinical malaria and subclinical Plasmodium infections in Pailin Province, Cambodia. *Heliyon* 2017; **3**(11): e00447.

4. Pava Z, Burdam FH, Handayuni I, et al. Submicroscopic and Asymptomatic Plasmodium Parasitaemia Associated with Significant Risk of Anaemia in Papua, Indonesia. *PloS one* 2016; **11**(10): e0165340.

5. Baidjoe AY, Stevenson J, Knight P, et al. Factors associated with high heterogeneity of malaria at fine spatial scale in the Western Kenyan highlands. *Malaria journal* 2016; **15**: 307.

6. da Silva NS, da Silva-Nunes M, Malafronte RS, et al. Epidemiology and control of frontier malaria in Brazil: lessons from community-based studies in rural Amazonia. *Transactions of the Royal Society of Tropical Medicine and Hygiene* 2010; **104**(5): 343-50.

7. Ladeia-Andrade S, Ferreira MU, de Carvalho ME, Curado I, Coura JR. Age-dependent acquisition of protective immunity to malaria in riverine populations of the Amazon basin of Brazil. *The American journal of tropical medicine and hygiene* 2009; **80**(3): 452-9.

8. Tadesse FG, Slater HC, Chali W, et al. The relative contribution of symptomatic and asymptomatic Plasmodium vivax and Plasmodium falciparum infections to the infectious reservoir in a low-endemic setting in Ethiopia. *Clinical infectious diseases : an official publication of the Infectious Diseases Society of America* 2018.

9. Eisele TP, Keating J, Bennett A, et al. Prevalence of Plasmodium falciparum infection in rainy season, Artibonite Valley, Haiti, 2006. *EID* 2007; **13**(10): 1494-6.

10. Fontoura PS, Finco BF, Lima NF, et al. Reactive Case Detection for Plasmodium vivax Malaria Elimination in Rural Amazonia. *PLoS neglected tropical diseases* 2016; **10**(12): e0005221.

11. Cerutti C, Jr., Boulos M, Coutinho AF, et al. Epidemiologic aspects of the malaria transmission cycle in an area of very low incidence in Brazil. *Malaria journal* 2007; **6**: 33.

12. Alves J, Roque AL, Cravo P, et al. Epidemiological characterization of Plasmodium falciparum in the Republic of Cabo Verde: implications for potential large-scale re-emergence of malaria. *Malaria journal* 2006; **5**: 32.

13. Hustedt J, Canavati SE, Rang C, et al. Reactive case-detection of malaria in Pailin Province, Western Cambodia: lessons from a year-long evaluation in a pre-elimination setting. *Malaria journal* 2016; **15**(1): 132.

14. Gomez MK, Caicedo MA, Gaitan A, et al. Characterizing the malaria rural-to-urban transmission interface: The importance of reactive case detection. *PLoS neglected tropical diseases* 2017; **11**(7): e0005780.

15. Hsiang MS, Ntshalintshali N, Kang Dufour MS, et al. Active case-finding for malaria: A three-year national evaluation of optimal approaches to detect infections and hotspots through reactive case detection in the low transmission setting of Eswatini. *Clinical infectious diseases : an official publication of the Infectious Diseases Society of America* 2019.

16. van Eijk AM, Ramanathapuram L, Sutton PL, et al. What is the value of reactive case detection in malaria control? A case-study in India and a systematic review. *Malaria journal* 2016; **15**(1): 67.

17. Zelman BW, Baral R, Zarlinda I, et al. Costs and cost-effectiveness of malaria reactive case detection using loop-mediated isothermal amplification compared to microscopy in the low transmission setting of Aceh Province, Indonesia. *Malaria journal* 2018; **17**(1): 220.

18. Herdiana H, Cotter C, Coutrier FN, et al. Malaria risk factor assessment using active and passive surveillance data from Aceh Besar, Indonesia, a low endemic, malaria elimination setting with Plasmodium knowlesi, Plasmodium vivax, and Plasmodium falciparum. *Malaria journal* 2016; **15**: 468.

19. Stevenson JC, Stresman GH, Baidjoe A, et al. Use of different transmission metrics to describe malaria epidemiology in the highlands of western Kenya. *Malaria journal* 2015; **14**(1): 418.

20. Bousema T, Stresman G, Baidjoe AY, et al. The impact of hotspot targeted interventions on malaria transmission in Rachuonyo South district in the western Kenyan Highlands: A cluster-randomized controlled trial. *PLoS medicine* 2016.

21. Aidoo EK, Afrane YA, Machani MG, et al. Reactive case detection of Plasmodium falciparum in western Kenya highlands: effective in identifying additional cases, yet limited effect on transmission. *Malaria journal* 2018; **17**(1): 111.

22. Pongvongsa T, Nonaka D, Iwagami M, et al. Household clustering of asymptomatic malaria infections in Xepon district, Savannakhet province, Lao PDR. *Malaria journal* 2016; **15**(1): 508.

23. Adhikari B, Phommasone K, Pongvongsa T, et al. Factors associated with population coverage of targeted malaria elimination (TME) in southern Savannakhet Province, Lao PDR. *Malaria journal* 2017; **16**(1): 424.

24. Landier J, Parker DM, Thu AM, et al. Effect of generalised access to early diagnosis and treatment and targeted mass drug administration on Plasmodium falciparum malaria in Eastern Myanmar: an observational study of a regional elimination programme. *The Lancet* 2018; **391**(10133): 1916-26.

25. Parker DM, Landier J, Thu AM, Lwin KM, Delmas G, Nosten FH. Scale up of a Plasmodium falciparum elimination program and surveillance system in Kayin State, Myanmar. *Wellcome Open Research* 2017; **2**(96).

26. McCreesh P, Mumbengegwi D, Roberts K, et al. Subpatent malaria in a low transmission African setting: a cross-sectional study using rapid diagnostic testing (RDT) and loop-mediated isothermal amplification (LAMP) from Zambezi region, Namibia. *Malaria journal* 2018; **17**(1): 480.

27. Smith JL, Auala J, Tambo M, et al. Spatial clustering of patent and sub-patent malaria infections in northern Namibia: Implications for surveillance and response strategies for elimination. *PloS one* 2017; **12**(8): e0180845.

28. Naeem MA, Ahmed S, Khan SA. Detection of asymptomatic carriers of malaria in Kohat district of Pakistan. *Malaria journal* 2018; **17**(1): 44.

29. Carrasco-Escobar G, Gamboa D, Castro MC, et al. Micro-epidemiology and spatial heterogeneity of P. vivax parasitaemia in riverine communities of the Peruvian Amazon: A multilevel analysis. *Sci Rep* 2017; **7**(1): 8082.

30. Donald W, Pasay C, Guintran JO, et al. The Utility of Malaria Rapid Diagnostic Tests as a Tool in Enhanced Surveillance for Malaria Elimination in Vanuatu. *PloS one* 2016; **11**(11): e0167136.

31. Imwong M, Nguyen TN, Tripura R, et al. The epidemiology of subclinical malaria infections in South-East Asia: findings from cross-sectional surveys in Thailand-Myanmar border areas, Cambodia, and Vietnam. *Malaria journal* 2015; **14**: 381.

32. Cook J, Grignard L, Al-Eryani S, et al. High heterogeneity of malaria transmission and a large sub-patent and diverse reservoir of infection in Wusab As Safil district, Republic of Yemen. *Malaria journal* 2016; **15**(1): 193.

33. Kobayashi T, Kanyangarara M, Laban NM, et al. Characteristics of Subpatent Malaria in a Pre-Elimination Setting in Southern Zambia. *The American journal of tropical medicine and hygiene* 2019; **100**(2): 280-6.

34. Stresman GH, Kamanga A, Moono P, et al. A method of active case detection to target reservoirs of asymptomatic malaria and gametocyte carriers in a rural area in Southern Province, Zambia. *Malaria journal* 2010; **9**: 265.

35. Deutsch-Feldman M, Hamapumbu H, Lubinda J, et al. Efficiency of a malaria-reactive test-an-treat program in southern Zambia: A prospective, observational study. *The American journal of tropical medicine and hygiene* 2018.
