# Supplementary material for: Quantifying Plasmodium falciparum infections clustering within households to inform household-based intervention strategies for malaria control programs: An observational study and meta-analysis from 41 malaria-endemic countries
Source: PLoS Med. 2020 Oct 29;17(10):e1003370. doi: 10.1371/journal.pmed.1003370 (PMC7595326; doi:10.1371/journal.pmed.1003370)
Supplement: S1 Fig — (DOCX) [file pmed.1003370.s007.docx]

**Supporting Figure 1: Flow diagram providing an overview of the results from the literature search to identify eligible studies included in the meta-analysis.**
