# Supplementary material for: Quantifying Plasmodium falciparum infections clustering within households to inform household-based intervention strategies for malaria control programs: An observational study and meta-analysis from 41 malaria-endemic countries
Source: PLoS Med. 2020 Oct 29;17(10):e1003370. doi: 10.1371/journal.pmed.1003370 (PMC7595326; doi:10.1371/journal.pmed.1003370)
Supplement: S3 Fig — Each panel shows the results of the simulated data for different transmission strata according to the PCR prevalence in a community of 1,000 people (e.g., 1% to 5%, >5% to 15%, and >15%). The height of each bar represents the total proportion of infections within a community, with the colour representing the proportion of infections that would be detected according to each strategy (and corresponding uncertainty) with the grey section showing the infections that would be missed. (DOCX) [file pmed.1003370.s009.docx]

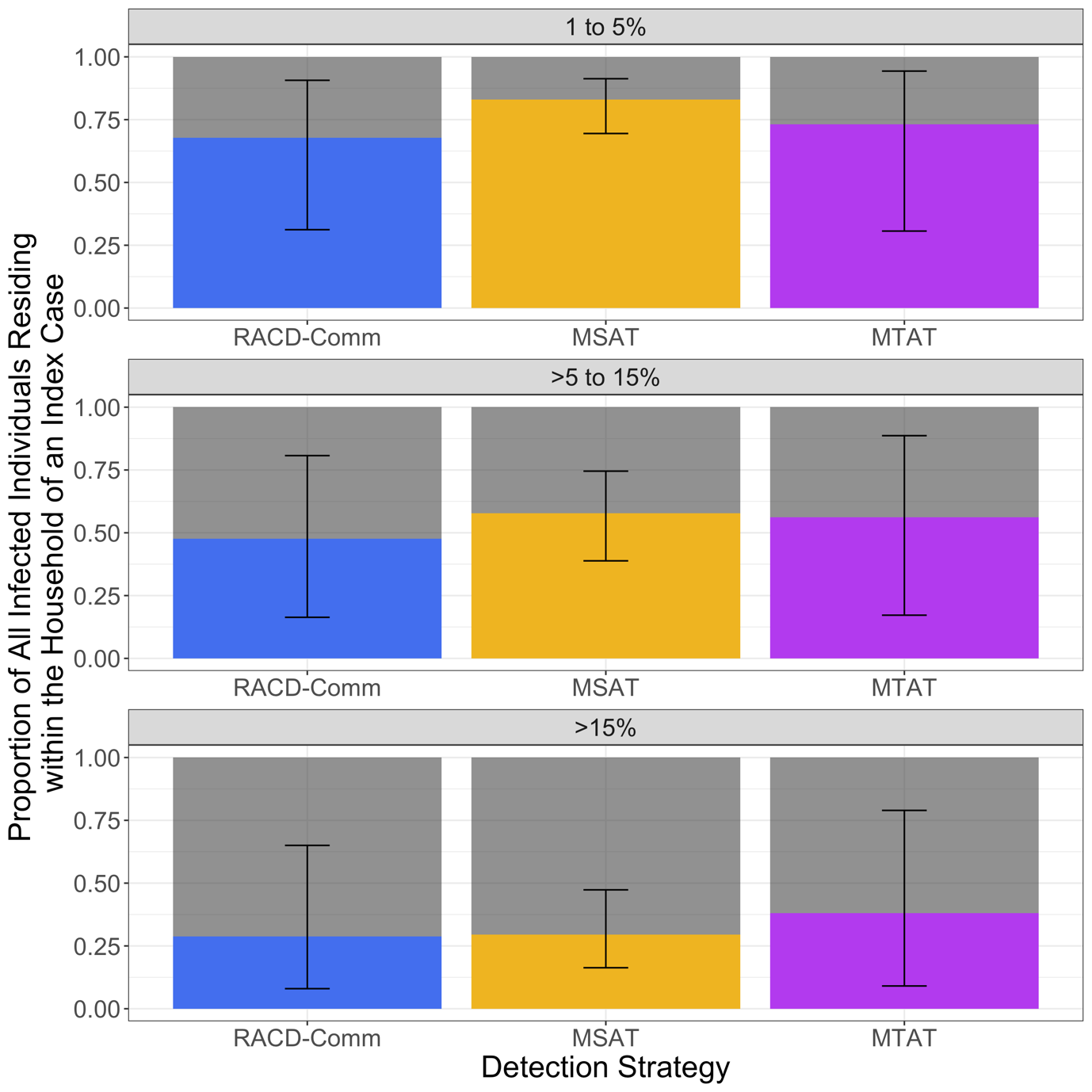


**Supporting Figure 3: The proportion of all infections in a community that would likely be detected if RACD, MSAT, or MTAT strategies would be employed by transmission strata.** Each panel shows the results of the simulated data for different transmission strata according to the PCR prevalence in a community of 1000 people (e.g. 1 to 5%, >5 to 15%, and >15%). The height of each bar represents the total proportion of infections within a community, with the colour representing the proportion of infections that would be detected according to each strategy (and corresponding uncertainty) with the grey section showing the infections that would be missed.
