# Supplementary material for: Quantifying Plasmodium falciparum infections clustering within households to inform household-based intervention strategies for malaria control programs: An observational study and meta-analysis from 41 malaria-endemic countries
Source: PLoS Med. 2020 Oct 29;17(10):e1003370. doi: 10.1371/journal.pmed.1003370 (PMC7595326; doi:10.1371/journal.pmed.1003370)
Supplement: Attachment — Submitted filename: ReviewerResponse_V2.1.docx [file pmed.1003370.s012.docx]

PLoS Medicine

Review Committee

July 11^th^, 2020

Dear Editor,

RE: Cover letter for revised manuscript submission (PMEDICINE-D-20-00366R1)

Thank you for considering our manuscript now titled ‘**Quantifying the clustering of *Plasmodium falciparum* infections within households to inform household-based intervention strategies for malaria control programs: A statistical and meta-analysis from 41 malaria endemic countries’** for publication in PLoS Medicine. We appreciate the feedback and the opportunity to revise our manuscript accordingly. We believe this revised version is much improved from the original submission and we would like to thank those who provided input for their valuable suggestions.

In summary,

- The editorial changes were completed as requested
- Text clarifying non-peri-domestic transmission was added
- Additional text to be clear about why the results of the DHS and meta-analysis were not quantitatively compared has been included.

Attached please find our detailed point-by-point response to each comment.

Please do not hesitate to contact us if you have any further questions.

Kind regards on behalf of all authors,

Gillian Stresman

London School of Hygiene & Tropical Medicine

Department of Immunology and Infection

Keppel Street, London UK, WC1E 7HT

[Gillian.Stresman@lshtm.ac.uk](mailto:Gillian.Stresman@lshtm.ac.uk)

Requests from the editors:

**Please revise your title according to PLOS Medicine's style. Your title must be nondeclarative and not a question. It should begin with main concept if possible. "Effect of" should be used only if causality can be inferred, i.e., for an RCT. Please place the study design ("A randomized controlled trial," "A retrospective study," "A modelling study," etc.) in the subtitle (ie, after a colon). Please also add a country setting.**

*As requested, we have revised the manuscript title to conform to PLoS Medicine’s guidelines. The new title is*

*LL1-4: “*Quantifying the clustering of *Plasmodium falciparum* infections within households to inform household based intervention strategies for malaria control programs: A statistical analysis of Demographic Health and Surveillance data from 23 African countries and a meta-analysis of studies from 20 malaria endemic countries*”*

**In the abstract:**

**please indicate dates around the data sets and cohort analysis and also some geographical information.**

*The dates and locations of the data used in the analysis have been added to the abstract as requested. Specifically, the DHS surveys were conducted between 2006 and 2018 in African countries whereas the data used for the meta-analysis included data from 20 countries between 2006 and 2019, originating in 20 countries across all malaria endemic regions (e.g. Africa, South America, Middle East, and Southeast Asia).*

LL49-53: “Data included 59,050 infections in 208,140 children under 7 years old by microscopy/RDT from 57 DHS surveys conducted between 2006 and 2018 from 23 African countries and 11,349 infections across all ages detected by molecular tools in 132,590 individuals in 43 studies between 2006 and 2019 in 20 countries across all malaria endemic regions obtained…”

**We in addition need summary demographic information. “;**

*Whilst we included basic (age, sex) demographic data in the analysis with the DHS datasets, a similar level of granularity was not extractable from literature for the meta-analysis. We wanted to ensure that the presentation of results from the two different datasets (DHS & studies extracted from literature) were as comparable as possible, so we haven’t included any of the summary demographic information. We therefore feel that providing summary demographic information would not be too useful for the reader, however, we will follow editorial advice here and can include this information if considered useful.*

**Please add p values along with the provided 95% Cis here in the abstract and throughout including tables.**

*The corresponding p-values have been added to the text at all instances, including presenting those for the difference in magnitude of clustering between strategies.*

**The final sentence of the Methods and Findings section of the abstract should include a sentence or two on the limitations of the study.**

*The following sentence has been added to the methods and finding section of the abstract to highlight some of the key limitations.*

*LL 63-68: “*However, our analysis did not account for any malaria control interventions in place, malaria seasonality or the likely heterogeneity of transmission within study sites. Clustering may thus have been underestimated.”

**At this stage, we ask that you include a short, non-technical Author Summary of your research to make findings accessible to a wide audience that includes both scientists and non-scientists. The Author Summary should immediately follow the Abstract in your revised manuscript. This text is subject to editorial change and should be distinct from the scientific abstract. Please see our author guidelines for more information:** [**https://journals.plos.org/plosmedicine/s/revising-your-manuscript#loc-author-summary**](https://protect-eu.mimecast.com/s/jpbeCp215inMD8RTqb8Si?domain=journals.plos.org)

*The non-technical Author Summary has been included after the abstract, as requested.*

*LL 76-111: “***Author Summary**

**Why was this study done?**

- Malaria is a vector-borne parasitic infection that results in both symptomatic and asymptomatic infections and is a particularly important problem in African, South American and Southeast Asian countries.
- When malaria transmission becomes low, malaria infections tend to become clustered within populations. In such situtaions, malaria programs can refine their strategies and begin to target malaria interventions specifically to include household members of malaria-infected individuals detected at the health facility, through community screening of febrile individuals or through mass-testing individuals for malaria.
- To make informed decisions on if, and when, programs should consider household-targeted malaria interventions, evidence is needed on whether malaria infections consistently cluster in households and which strategy is best able to target asymptomatic infections.

**What did the researchers do and find?**

- We analysed data from 208,140 African children collected from demographic health surveillance surveys between 2006 and 2018 and conducted a meta-analysis of 132,590 individuals of all ages from all malaria endemic settings around the world collected between 2006 and 2019.
- Both datasets show that malaria infections do cluster in households at all transmission intensities, but clustering becomes more pronounced as transmission intensity declines (i.e a larger proportion of infected individuals within a population are clustered in fewer households).
- If all household members of index cases were targeted with interventions, they could potentially treat ~75% of all infections in a community once transmission intensity is very low.

**What do these findings mean?**

- In locations where local malaria transmission occurs , malaria infections cluster within households of index cases, regardless of how that index case was identified.
- Household targeted strategies, for instance giving all residents of index households a curative dose of an effective anti-malarial drug, provide malaria control programs with an option to easily target infections that otherwise may not be detected.
- In the future, understanding how reactive strategies contribute to achieving malaria elimination will be important in identifying the best strategy and for determining how long it should be sustained. “

**Did your study have a prospective protocol or analysis plan? Please state this (either way) early in the Methods section. a) If a prospective analysis plan (from your funding proposal, IRB or other ethics committee submission, study protocol, or other planning document written before analyzing the data) was used in designing the study, please include the relevant prospectively written document with your revised manuscript as a Supporting Information file to be published alongside your study, and cite it in the Methods section. A legend for this file should be included at the end of your manuscript. B ) If no such document exists, please make sure that the Methods section transparently describes when analyses were planned, and when/why any data-driven changes to analyses took place. c) In either case, changes in the analysis-- including those made in response to peer review comments-- should be identified as such in the Methods section of the paper, with rationale.**

*A protocol or analytical plan was not prospectively published, either as a published manuscript, or a funding or ethics application. This has been clearly stated in the methods section and text amended to transparently describe the planning of activities and any changes that occurred as part of implementation or associated with the reviewers comments.*

LL190-193 “A review protocol or analysis plan was not prospectively published, but is reported in full here, including any adaptations that took place..”

LL245-249: “We intended to look at clustering in areas with non-peri-domestic transmission (e.g. forest goers, as defined by the study authors) but only two eligible studies, both conducted in Cambodia in populations near forests that explicitely studied forest transmission, were identified. For clarity of message, this demographic group was removed from the analysis.

**It seems this analysis is using data from Children, this needs to be added to the title, if so, the abstract and made explicitly clear in the main text – it’s not until Table 1 that this was apparent to me. The reader needs to understand this from the start. It is slightly confusing atm as you then say the malaria status of all household members are tested. If this is not the main part of the analysis, please be explicit.**

*Thank you for highlighting this point. The editor is correct in that the DHS data included only children tested for malaria whereas the data used as part of the meta-analysis included all residents within households therefore both children and adults were tested for malaria. The abstract, and main text have been amended to clarify this point as per the comment above.*

**There is a lack of relevance in the main text about the locations and geographic regions that are being surveyed. Please add.**

*The DHS data all originated in African countries whereas the data extracted from the literature for the meta-analysis was not limited by region with studies included from south American, African, Middle East and southeast Asian settings. The geographic regions where the data originated have been added throughout.*

*LL196: “…*data from 57 DHS surveys from 23 African countries containing…”

LL228: “…and were published from inception to August 1, 2019, no limitation to geographic region, and with results…”

LL286: “…surveys spanning 23 African countries, we extracted data…”

LL312-314: “The included studies were predominantly conducted in African (17) or Asian (15) settings with 8 from South America, 2 in the Pacific and 1 from the Middle Eastern regions.”

**Was written consent provided?**

*Written informed consent was provided for the data collection and described in the original study publications, with references provided in the supplemental text 4. Text has been added to the methods section to clarify this point.*

*LL 202-203: “*Informed parental consent was obained as per the approved DHS study protocols.”

*LL 235-236: “*Informed consent for data collection was obtained as per the original data collection protocol as outlined in the respective publication (S4 Text).”

**Please provide a table with demographic information (main text)**

*Although the information is also available in the supplementary text, this table has also been added to the main text including the summary information relevant to the analysis that was conducted for both the DHS data and studies included in the meta-analysis.*

*LL 302-305:*

| Table 2: Summary information providing an overview of the data included in the data analysis using the DHS data and published literature included in the meta-analysis. | | |
| --- | --- | --- |
|  | ***DHS Analysis Data*** | ***Meta-Analysis Data*** |
| N Studies | 57 | 43 |
| Year Data Collection (range) | 2006-2018 | 2006-2019 |
| Continental Region N - (n unique countries) | | |
| African | 57 (23) | 17 (7) |
| Americas | 0 | 8 (4) |
| Asia | 0 | 15 (7) |
| Pacific and Middle East | 0 | 3 (3) |
| All age surveys vs children only -% (n/N studies) | 10.5% (6/57) | 97.7% (42/43) |
| N Individuals Sampled (n malaria positive) | 420,659 (59,050) | 132,590 (11,349) |
| Number of Individuals Per Model Type (n malaria positive) | | |
| RACD vs Community | 72,498 (24,836) | 5,661 (288) |
| RACD vs Neighbours | 0 | 23,497 (564) |
| MSAT | 177,243 (50,590) | 40,851 (6,238) |
| MTAT | 208,140 (59,050) | 62,581 (4,259) |

Please ensure that the study is reported according to the STROBE guideline, and include the completed STROBE checklist as Supporting Information. 1 Please add the following statement, or similar, to the Methods: "This study is reported as per the Strengthening the Reporting of Observational Studies in Epidemiology (STROBE) guideline (SChecklist)." The STROBE guideline can be found here: [http://www.equator-network.org/reporting-guidelines/strobe/](https://protect-eu.mimecast.com/s/lS-ICqZ14t80W5MU7fUXU?domain=equator-network.org) When completing the checklist, **please use section and paragraph numbers**, rather than page numbers.

*The STROBE checklist included as Supplemental Text 3 has been updated based on the revised text and included section and paragraph numbers rather than simply page numbers.*

Comments from the reviewers:
Reviewer #1: The manuscript by Stresman et al. "Do Plasmodium falciparum infections cluster at the household level? A meta-analysis of the evidence to inform household based intervention strategies for malaria control programs"

Major

**I full-heartedly agree with authors' claim that "Reactive malaria strategies are predicated on the assumption that individuals infected with malaria are clustered within households or neighbourhoods. Despite the widespread programmatic implementation of such strategies, little empirical evidence exists as to whether such strategies are appropriate and if so, how they should be most effectively implemented." Unfortunately, authors fail to provide information that were not found in the previous studies or give different angle to the issue. 

I do not think there is a single intervention that works on its own, and household- based intervention strategies are not exception. It is yet unclear to me at what level, or stage of transmission intensity, household-based intervention can actually contribute to elimination. Different approaches such as mass drug administration (The Gambia. Mwesigwa et al., 2019), or indoor residual spraying (Uganda. Nankabirwa et al., 2019) that are more intensive seem to result in short lasting effect.**

*We thank the reviewer for their comment, however as Reviewer 2 has noted, we believe that this work provides the first comprehensive and rigorous synthesis of data to support the notion that malaria infections, both those detectable by programmatic tools (RDT/microscopy as per the DHS data analysis) and those with lower density infections detectable only through molecular means (PCR/LAMP as per the literature included in the meta-analysis), cluster within households. Here we take a step back and confirm the assumptions driving both research and programmatic activities that household clustering is a consistent facet of malaria transmission at all transmission intensities. Furthermore, we extend this thinking and assess the efficacy of programmatic means for detecting asymptomatic infections. The question of whether household targeted activities contribute to malaria elimination is an important one, but before that can be addressed, the question of whether infections cluster where programmatically detectable ones originate, is the important first step to confirm. The cited studies (as well as many others), indeed highlight the short-lived impact of many interventions. We agree that there is a necessity for long-term interventions to sustain the initial gains. Household targeting, very different from for instance the single round MDA in The Gambia in Mwesigwa et al., is an approach that is considered operationally sustainable when transmission declines. The efforts required will decline when the number of malaria cases (continue to) decline. Further text has been added to the discussion to highlight this important point.*

*LL371-376: “Interventions targeting both entire communities or households known to have malaria infections have resulted in short-lived reductions in malaria transmission. [22, 23] However, strategies targeting household is considered to be operationally sustainable once transmission is low. Given that achieving elimiantion will likely require a sustained effort over the long-term, reactive strategies targeting households provides a viable programmatic option.“*

**Line 102-4 "However, there is currently little evidence as to whether household clustering is a consistent feature of malaria micro-epidemiology and whether the extent of this clustering (which would determine the fraction of the undetected reservoir that could be targeted) justifies the use of household-based reactive strategies." - I thought clustering at different socio-spatial level, such as household, is indeed a part of malaria micro-epidemiology…?** **Readers may benefit from some clarification with what you are referring with malaria micro-epidemiology in this sentence.** 

*The text has been modified to clarify what we meant by micro-epidemiology:*

*LL 156-157: “….*feature across settings with different malaria epidemiological conditions (e.g. vector species, population densities etc.) and…”

**Line 362 "if a sufficient proportion of all infections can be targeted." - I think this is the fundamental question we are all interested in, but I did not see this aspect discussed in discussion**.

*We agree with the reviewer in that we are ultimately interested in whether such strategies can contribute to and/or accelerate malaria elimination. However, the aim of this work was to focus on the more fundamental question of confirming the assumptions that infections cluster within households of programmatically detected infections. We then extended this to demonstrate what proportion of infections may be detected based on the evidence presented. Extending the discussion to include text as to whether such strategies would contribute to elimination would be speculative and not supported by the data available. To make the connection to this fundamental question we have extended the text to clarify the potential implications of our results on elimination in both the discussion and conclusion sections.*

*LL 395-398: “*Based on the results presented here, if effectively sustained over time, it is plausible that reactive strategies employing presumptive treatment of index households, could contribute to accelerating the road to elimination.”

LL 474-476: “Such activities have the potential to enhance elimination efforts and, in combination with effective vector control and strong health systems, may contribute to reducing transmission if a sufficient proportion of all infections can be targeted.”

**Minor**
**Using antimalarials use as a proxy for seeking care at health facilities - In order for this assumption to work well, it is important that antimalarials are available at health facilities and not at private pharmacies**. **Among 23 countries DHS surveys were obtained from, do you have information on how antimalarials are distributed**?

*The reviewer is correct in that using antimalarial drugs as a proxy for care seeking behaviour is an imperfect approach and will likely vary based on the health system in the specific country. However, based on the limited data routinely collected as part of the DHS surveys, and what was consistently collected across studies, we considered this as the best proxy to define the programmatic detection models. However, despite the limitations with the data available, given that the results were consistent between the DHS and meta-analysis, this suggests that the limitation is not likely to qualitatively change the findings. The discussion text has been adapted to ensure this limitation is clearly stated.*

*LL 440-442: “…*with strategy definitions modified based on available variables, some of which acted as imperfect proxy indicators, routinely collected as part of the DHS…”

**Figure 2 - Please use image with higher resolution**

*A revised Figure 2 has been included in the submission with the resolution increased from 300 to 500 dpi.*

**Line 326: "were" should be "where"?** 
*Corrected*

Reviewer #2: Dear Authors,
In this paper the authors investigated to what extent Plasmodium falciparum infections cluster at the household level based on existing literature data for both patent and sub-patent infections. 
The review was conducted in a comprehensive way and collates evidence which indicates to what extent the crucial, but not yet rigorously tested, assumption of clustering of malaria positive individuals at the household level, holds. National Malaria programs in different countries include spatially targeted interventions based on the assumption that indeed some form of spatial clustering at the household (or neighboring households) is present. The data demonstrated extensive clustering for both patent and sub-patent infections at the household level and increasing odds of finding positive cases at index households with decreasing prevalence. Moreover they point out that in areas where malaria is peri-domestic the existing programmatic options are sufficient to identify those households where residuals infections are likely to be found. They suggest that once identified treating all members of such index or 'source' households could provide an alternative to mass treatment campaigns and potentially enhance elimination efforts. 
In summary I think the authors did an excellent job and the paper certainly fits the criteria for publication in PloS Medicine.

However I would like to point out two major aspects which could warrant some more discussion. 

**Major comments:**
**Peri-domestic transmission is somewhat loosely defined as malaria transmission which occurs around the household. The authors point out in their discussion that peri-domestic transmission is common in the African setting, but not always so in other places. For example in a Cambodian forest setting transmission can occur far away from home (typically associated with a dual housing setting). The authors did not emphasis in the main text that the entire DHS survey is compiled of countries from the African continent, whereas the PCR/LAMP meta-analysis is a mix of countries from different regions.**

*Thank you for your comments. As highlighted above, we have included the new table 2 including data summarizing the different studies included in the analysis, as well as text throughout to highlight that the DHS data all originated in the African region as per the point below. Furthermore, text to clarify and qualify the definitions of peri-domestic vs. non peri-domestic transmission has been added.*

*LL 50-53: “…*DHS surveys conducted between 2006 and 2018 from 23 African countries and 11,349 infections across all ages detected by molecular tools in 132,590 individuals in 43 studies between 2006 and 2019 in 20 countries across all malaria endemic regions obtained …”

*LL116-117: “..*(i.e. contact with the mosquito vector occurs around the household considered to be the main residence),…”

*LL 196: “…*from 57 DHS surveys from 23 African countries containing…”

LL286: “…surveys spanning 23 African countries..”

LL312-314: “The included studies were predominantly conducted in African (17) or Asian (15) settings with 8 from South America, 2 in the Pacific and 1 from the Middle Eastern regions (Table 2).”

**They did state however on line 177 that they retained those studies which were expected to have peri-domestic transmission, and also in table 2 that 'region' didn't affect the results. However, it is unclear how these choices were made, what exactly was their basis for deciding that transmission is expected to be peri-domestic (or not)? Or perhaps more informative which studies were not retained on the basis of expecting non peri-domestic transmission. This info could be included in the supp material, e.g. the flowchart of the meta-analysis, extended figure 3.**

*The reviewer makes an excellent point in that we did not explicitly define criteria around determining whether a setting could be considered as having peri-domestic transmission. We have amended the text to further clarify that peri-domestic transmission is considered as when transmission is expected to occur in the household considered to be the main residence and delineating study sites as peri or non-peri domestic was based on the definition used in the original publication.*

*LL245-249: “*We intended to look at clustering in areas with non-peri-domestic transmission (e.g. forest goers, as defined by the study authors) but only two eligible studies, both conducted in Cambodia in populations near forests that explicitely studied forest transmission, were identified. For clarity of message, this model group was removed from the analysis.*”*

*LL457-462: “*Next, our definition of peri-domestic transmission was based on the epidemiological conditions as reported by the authors of the original publication. It is possible that included studies were misclassified as being peri-domestic transmission. However, impact of misclassification would be expected to attenuate the pooled OR and therefore is expected to have minimal impact on the interpretation of the results.”

**It would be of interest from a more programmatic point of view to see whether or not these omitted studies are all from one country, one continent, … as well as some additional info on what they do consider as peri-domestic and what not. For example in Cambodia, farmers often have one residence in the village and an additional small house several km away at their field plots, in which they also spend the nights, especially when crop planting/harvesting needs to be done. This system is much more outspoken in the north-east of Cambodia versus the western provinces (Pailin, Battambang), but to my knowledge, not absent. So on what basis could a program manager decide that his/her setting is sufficiently 'peri-domestic' to initiate a spatial intervention based on index households determined from programmatic info available?**

*The reviewer is correct in that the excluded studies due to peri-domestic transmission were all from the same setting: Cambodia. The results were as expected with the odds of infections in co-travellers being 9.19 times higher than randomly selected households within their community of residence or 2.2 times higher than those residing within the same household as the index case. Delineating peri-domestic vs. other transmission is an important distinction from a programmatic perspective and providing insight on how this can be done is beyond the scope of this paper. However, the more explicit definition provided as per the comment above, may be useful for programs to consider. Text has been added to clarify the notion that the local transmission context is what’s important and does vary within countries so a blanket strategy may not be possible.*

*LL431-433: “…t*he local transmission context within countries (e.g. within Cambodia, both household and forest transmission occurs, depending on the area within the country) [14].”

**The second point I want to raise is regarding the differences in odds ratio of the patent versus sub- patent infections. As the DHS survey is compiled of African countries, generally having higher prevalence rates, while the sub-patent data are comprised of countries on three continents having generally lower prevalence, what could have driven a two- to nearly three-fold increase in odds ratio ('clustering') in the DHS data versus the PCR/LAMP data? Does this simply reflects the way the analysis is performed or could there be other reasons? Perhaps the authors could comment on this apparent contradiction in the text as well.** 

*We agree with the reviewer in that the magnitude of the results in the DHS data analysis is much higher than that observed in the results of the meta-analysis. The differences could be related to many factors, and are likely due to a combination of the different populations sampled (children vs all ages), how ‘index’ households were defined based on the data available, the malaria diagnostic methods used (RDT vs molecular), the study setting and the differences in transmission intensity in African vs other regions, the vector populations, the analytical methods used etc. PCR/LAMP detected infections are also plausible older than those detected in the DHS system (by RDT or microscopy).*

*Considering these uncertainties, we have avoided any direct comparison of the magnitude of clustering and instead make conclusions based on qualitative comparisons, specifically that both analyses show similar results with increasing magnitude of clustering in households of detectable infections. We had added some text to emphasize this point and the limitations inhibiting direct comparisons between the datasets.*

LL366-367: “Although the differences in the data collection protocols and analytical methods used precludes a direct comparison, together our results suggest that in…”

LL453-448: “The differences between the DHS and published literature aggregated to conduct the meta-analysis in terms of target population, data available, geographic spread, and analytical methods employed were such that a direct comparison between the resulting ORs is not meaningful. However, given the trends of increasing clustering as transmission decreased were qualitatively similar, the analysis is nevertheless informative.”

**Minor comments.**
**L39: typo: detection instead of detecation**

*Corrected*

**L136: It is striking that all data collected from 23 DHS surveys are all countries from the African continent, therefor add '23 African countries' here.**

*Corrected*

**L177: On what basis exactly was a study designated as being non peri-domestic, and how many such studies were excluded?**

*As per the comment above, studies were designated as non peri-domestic transmission based on the description of the study setting in the original publication. As was outlined in Supplemental figure 3, 2 studies were excluded for this reason. The text has also been amended to clarify this exclusion criteria.*

*LL245-249: “*We intended to look at clustering in areas with non-peri-domestic transmission (e.g. forest goers, as defined by the study authors) but only two eligible studies, both conducted in Cambodia in populations near forests that explicitely studied forest transmission, were identified. For clarity of message, this model group was removed from the analysis.*”*

**L151: Define the uninformative priors in the S2 text exactly, what distribution was used?**

*The text in S2 has been updated to include the specific details on the priors used.*

*S2-Text: “*Normal distributions with mean 0 and standard deviation 15”

**L156 & S2 text: Did you truly fit a no-intercept regression? If not add 'beta 0' as intercept coefficient. In fact I would suggest you simply extract the STAN code, and add it in S2 text, it will become clear immediately how the model was fitted (as well as what priors were used).**

*The text in S2 has been amended accordingly and the STAN code has been added as requested.*

*S2-Text:*

*“* $\alpha_{k}$ is an intercept within the regression framework used.”

*“***STAN Code for the Model Used**

**data {**

int<lower=0> N;

vector[N] Overall_Prevalence;

vector[N] Other_HH_Member_Status;

vector[N] Num_Respondents;

int Infection_Status[N];

**}**

**transformed data {**

vector[N] interaction;

interaction = Overall_Prevalence .* Other_HH_Member_Status;

**}**

**parameters {**

real intercept;

real overall_prev_coef;

real other_hh_member_coef;

real interaction_coef;

real num_resp_coef;

**}**

**model {**

intercept ~ normal(0, 15);

overall_prev_coef ~ normal(0, 15);

other_hh_member_coef ~ normal(0, 15);

interaction_coef ~ normal(0, 15);

num_resp_coef ~ normal(0, 15);

Infection_Status ~ bernoulli_logit(intercept + overall_prev_coef * Overall_Prevalence + other_hh_member_coef * Other_HH_Member_Status + interaction_coef * interaction + num_resp_coef * Num_Respondents);

**}**

*“*

**L157/158: Upon first read it is simply not clear why you use an individual-level approach for the DHS data, but need a meta-analysis for the sub-patent infections of the PCR/LAMP data. I suggest to clarify this briefly in one/two sentences just before L158.**

*Individual-level data analysis was not possible as part of the meta-analysis because this level of data was not available for the majority of data, either because it was extracted directly from the literature, or because that was the condition with which authors were willing to share the data. The text has been modified to clarify this.*

*LL252-254: “*Individual-level data was not available for several studies, either on condition of data sharing or because it was extracted directly from the literature so the meta-analysis focused on the summary estimates per site.*”*

**L169: should be RDT or PCR, instead of AND?**

*The text has been amended to ensure clarity and emphasize the all household members had to be tested by molecular methods and a programmatic tool, here RDT or microscopy, had to be employed on the same household to define the index status according to one of the reactive models being tested (i.e. all residents, febrile residents only, or the clinical case only)*

**L197: prevalence instead of prevalanece**

*Corrected*

**L207: add 'African' countries**

*Corrected*

**L213: CI is here a credible interval, not a confidence interval given you did use MCMC. However on the same page L192 you mention confidence interval. You need to distinguish between the two as they, from a statistical point of view, are not the same.**

*The abbreviations have been amended throughout to reflect the difference between Credible (CrI) and Confidence (CI) intervals.*

**L244 - table 2: With region it seems the author indicate 'continent', or 'continental region', please adapt.**

*The text has been amended to reflect continental region, as suggested.*

**L357: individuals misspelled.**

*Corrected*

**L416: type: fine not find-scale**
*Corrected*


Reviewer #3: **I confine my remarks to statistical aspects of this paper. These were well done and I recommend publication.**

*Thank you for your comments.*
