# Supplementary material for: Quantifying Plasmodium falciparum infections clustering within households to inform household-based intervention strategies for malaria control programs: An observational study and meta-analysis from 41 malaria-endemic countries
Source: PLoS Med. 2020 Oct 29;17(10):e1003370. doi: 10.1371/journal.pmed.1003370 (PMC7595326; doi:10.1371/journal.pmed.1003370)
Supplement: Attachment — Submitted filename: ReviewerResponse3.2.docx [file pmed.1003370.s013.docx]

PLoS Medicine

Review Committee

September 8^th^, 2020

Dear Editor,

RE: Cover letter for revised manuscript submission (PMEDICINE-D-20-00366R1)

Thank you for considering our manuscript titled ‘**Quantifying the clustering of *Plasmodium falciparum* infections within households to inform household-based intervention strategies for malaria control programs: A statistical and meta-analysis from 41 malaria endemic countries’** for publication in PLoS Medicine. We appreciate the feedback and the opportunity to revise our manuscript accordingly. We believe this revised version is much improved from the original submission and we would like to thank those who provided input for their valuable suggestions.

In summary,

- The editorial changes were completed as requested

Attached please find our detailed point-by-point response to each comment.

Please do not hesitate to contact us if you have any further questions.

Kind regards on behalf of all authors,

Gillian Stresman

Assistant Professor

London School of Hygiene & Tropical Medicine

Department of Immunology and Infection

Keppel Street, London UK, WC1E 7HT

[Gillian.Stresman@lshtm.ac.uk](mailto:Gillian.Stresman@lshtm.ac.uk)

**EDITORIAL COMMENTS**

Can you please address the following issues as soon as possible? Your paper has been sent back to enable you to make these changes.

1. Please include the location from which you obtained the base map used in Fig 1 in the legends for this figure. Please note that, because all PLOS articles are published under a CC BY license ([creativecommons.org/licenses/by/4.0/](http://protect-eu.mimecast.com/s/P5gdCA1pkcNyoE5i2WLZL?domain=creativecommons.org)), we cannot publish proprietary maps such as Google Maps, Mapquest or other copyrighted maps. If your map was obtained from a copyrighted source please amend the figure so that the base map used is from an openly available source.

*The map was obtained at Natural Earth using the rnaturalearth R package. As per the terms of use, all maps are in the public domain. The following statement has been added to the legend text for Fig 1.*

*“*The world map was obtained from the rnaturalearch R package (V 0.1).”
